# Parliament and Publications

**Published:** 1911-03-11

**Authors:** 


					G92 HIE HOSPITAL March 11 "1911.
Parliament and Publications.
VACCINATION IN SCOTLAND.
In reply to a question asked by Sir Henry Crr.ik re to
-whether there had recently been a marked decline in the
percentage of infants vaccinated, and whether there had
l>een at the same time a marked increase in smallpox cases,
the Lord Advocate has furnished the following ctatlstical
information :?
Year. Births.
Statu orv
Pen enlace fleclara-
of infants 1 ions of
successfully conscien-
vaccinatecl. t.ious objec
tion.
1901 1^2,280 ?4.034
1902 132.360 i 85.203
1903 133 5<-3 85 443
1901 132 663 84.r/13
1905 131.4C3 fc5.14 7
1916 132 056 83.153
1907 128,691 ?5.e09
1908 131,406 69.399
1909 128,7,0 C5.286
253
7,258
15.F46
22,746
imM -pox
eases re-
ported to
Local
Gori'rn-
nent Hnar<
l.v-90
915
.^50
2 527
128
23
THE PLAGUE AND CHOLERA.
Sir E. Grey, in answer to a question asked by Mr.
Ginnell in the House of Commons, has stated that during
the continuance of any outbreak of plague, cholera, or
yellow fever, his Majesty's Consuls are expected to furnish
short telegraphic reports at least weekly, showing the
number of cases and deaths and the name of the locality
affected; in the case of the present epidemic in Manchuria
reports are received almost daily. His Majesty's Govern-
ment and most European countries are parties to inter-
national co-operation, the object of which is to apply in a
systematic manner the most modern methods of resisting
the spread of plague and cholera.
DECREASED BIRTH AND DEATH RATES.
The birth rate in England and Wales in 1910 was 24.8
pe~ 1,000 of the population, being 0.8 per 1,000 below the
vate in 1909 and lower than the rate in any other year on
record. The death rate in 1910 was 13.4 per 1,000 of the
population, being 1.1 per 1,000 below the rate in 1909, and
lower than the rate in any other year on record. The rate
of mortality among infants under one year of age to 1,000
registered births was 106, which is 3 per 1,000 below th8
rate in 1909 and lower than in any other year on record.
TROPICAL DISEASES.
The revenue of the Tropical Diseases Reserve Fund for
the year 1910 was ?3,245; the expenditure amounted to
?5,258 6s. 8d., and consisted of grants as follows :?Lon-
don School of Tropical Medicine, ?2,233 6s. 8d. ; Liverpool
School of Tropical Medicine, ?1,900; London University
?750; Cambridge University ?225; Dr. Sambin (in
connection with the Pellagra Research) ?150. In order to
meet the expenditure it was necessary to draw upon the
accumulated balance. In their report for the year 1910 the
Advisory Committee of the Fund state that they have noted
with interest the steps taken to combat malaria in the
tropical countries, and they refer to the valuable summary
of the question contained in Professor Ronald Ross' work
on the prevention of malaria published in 1910. The com-
mittee's report includes reports on anti-malarial measures
from Ceylon, Mauritius, Seychelles, Gold Coast, St. Lucien,
St. Vincent, Papua; reports from the Quick Laboratory,
Cambridge, and from the London and Liverpool Schools of
Tropical Medicine and reports on work done in Colonial
laboratories.?"Tropical Diseases Research Fund," Cd.
5,514, price Is. 6d.

				

